# High-fidelity is not superior to low-fidelity simulation but leads to overconfidence in medical students

**DOI:** 10.1186/s12909-019-1464-7

**Published:** 2019-01-21

**Authors:** Christina Massoth, Hannah Röder, Hendrik Ohlenburg, Michael Hessler, Alexander Zarbock, Daniel M. Pöpping, Manuel Wenk

**Affiliations:** Department of Anesthesiology and Intensive Care, University Hospital Münster, Albert-Schweitzer-Campus 1 (A1), 48149 Münster, Germany

**Keywords:** Simulation, Education, Medical, Overconfidence

## Abstract

**Background:**

Simulation has become integral to the training of both undergraduate medical students and medical professionals. Due to the increasing degree of realism and range of features, the latest mannequins are referred to as high-fidelity simulators. Whether increased realism leads to a general improvement in trainees’ outcomes is currently controversial and there are few data on the effects of these simulators on participants’ personal confidence and self-assessment.

**Methods:**

One-hundred-and-thirty-five fourth-year medical students were randomly allocated to participate in either a high- or a low-fidelity simulated Advanced Life Support training session. Theoretical knowledge and self-assessment pre- and post-tests were completed. Students’ performance in simulated scenarios was recorded and rated by experts.

**Results:**

Participants in both groups showed a significant improvement in theoretical knowledge in the post-test as compared to the pre-test, without significant intergroup differences. Performance, as assessed by video analysis, was comparable between groups, but, unexpectedly, the low-fidelity group had significantly better results in several sub-items. Irrespective of the findings, participants of the high-fidelity group considered themselves to be advantaged, solely based on their group allocation, compared with those in the low-fidelity group, at both pre- and post-self-assessments. Self-rated confidence regarding their individual performance was also significantly overrated.

**Conclusion:**

The use of high-fidelity simulation led to equal or even worse performance and growth in knowledge as compared to low-fidelity simulation, while also inducing undesirable effects such as overconfidence. Hence, in this study, it was not beneficial compared to low-fidelity, but rather proved to be an adverse learning tool.

## Background

Simulation is increasingly used for training and education of medical professionals [1].

Since its origins in the 1960s, with the development of simulation mannequins such as “ResusciAnne” or the “Harvey cardiology mannequin” for training of cardiological examination skills [2], simulation-based training has spread to various disciplines and remains a strongly growing market [3–7].

Numerous trials have amply demonstrated the positive effects of simulation-based training on technical skills, while also reducing peri-interventional risks and complications. These outcomes might translate into improved patient care [3, 6, 8, 9].

Simulation-based education seems to be ideal for providing medical students access to practical “hands-on” applications of their theoretical knowledge, by training of procedural skills and (single) tasks in simulated environments. This is widely received as a positive development, since a lack of practice remains a common complaint in medical education [10].

Due to the continuous technical development of hard- and software, current simulators provide a close-to-reality experience and contain features such as realistic physiological responses, the ability to communicate and interact with the mannequin, and various other feedback mechanisms. These highly realistic devices do not just function as single-task trainers, but present the user with complex and immersive scenarios by providing realistic feedback; and are therefore referred to as high-fidelity (HF) simulators. In contrast, part-task trainers with limited functions that meet only selected requirements for practicing procedural skills are referred to as low-fidelity (LF) simulators.

Intuitively, a positive correlation between the degree of realism of a simulator and the effect on learning outcomes of the trainees is assumed, but several studies have found no distinct advantage of HF compared to LF simulation with regards to improvement of knowledge or skills [11–13].

In a previous trial comparing the efficacy of simulation-based training versus problem-based discussions, we found no significant differences in short-term outcomes between groups for either theoretical or practical knowledge. However, we found significantly higher self-assessment scores and inflated self-confidence in the simulation-based training group, which profoundly overrating its abilities [14]. In educational programs, this is an undesirable effect, due to a positive link between overconfidence and risk-taking behavior [15, 16]. Some studies have shown that overconfidence is one of the most common cognitive biases leading to diagnostic errors [17, 18].

Whether simulation-based medical education per se favors the occurrence of inflated self-confidence and flawed self-judgment of individual skills, abilities and knowledge is not known. Hence, the aim of this trial was to examine the impact of HF versus LF simulation on self-assessment and confidence.

## Methods

### Study design

This randomized trial was conducted during a curricular advanced life support (ALS) training course session for medical students. The study was approved by the Ethics committee of the University of Münster (protocol number 2014–544-f-S) on 6 November 2014 and all students gave written informed consent to participate. All participants were misinformed regarding the real purpose of the study. Students were informed that a simple internal quality assessment of medical education and simulators was being carried out, but they were not informed that changes in confidence were to be assessed.

An a priori power calculation was conducted using the independent two-sample t-test, based on previously published data, to create a sufficient sample size in each group at a 1:1 ratio of controls (LF simulation) to experimental (HF simulation) subjects for independent groups, with a type I error of 0.05 and a power of 80%.

All fourth-year medical students were included in the study and were randomly distributed into 14 groups of 10 students each by the medical faculty of the University of Münster. These groups of students were then allocated to either a LF or a HF simulation group, using a randomization sequence with the method of permuted blocks.

### Survey of demographic data, theoretical knowledge and self-assessment

**Fig. 1:**
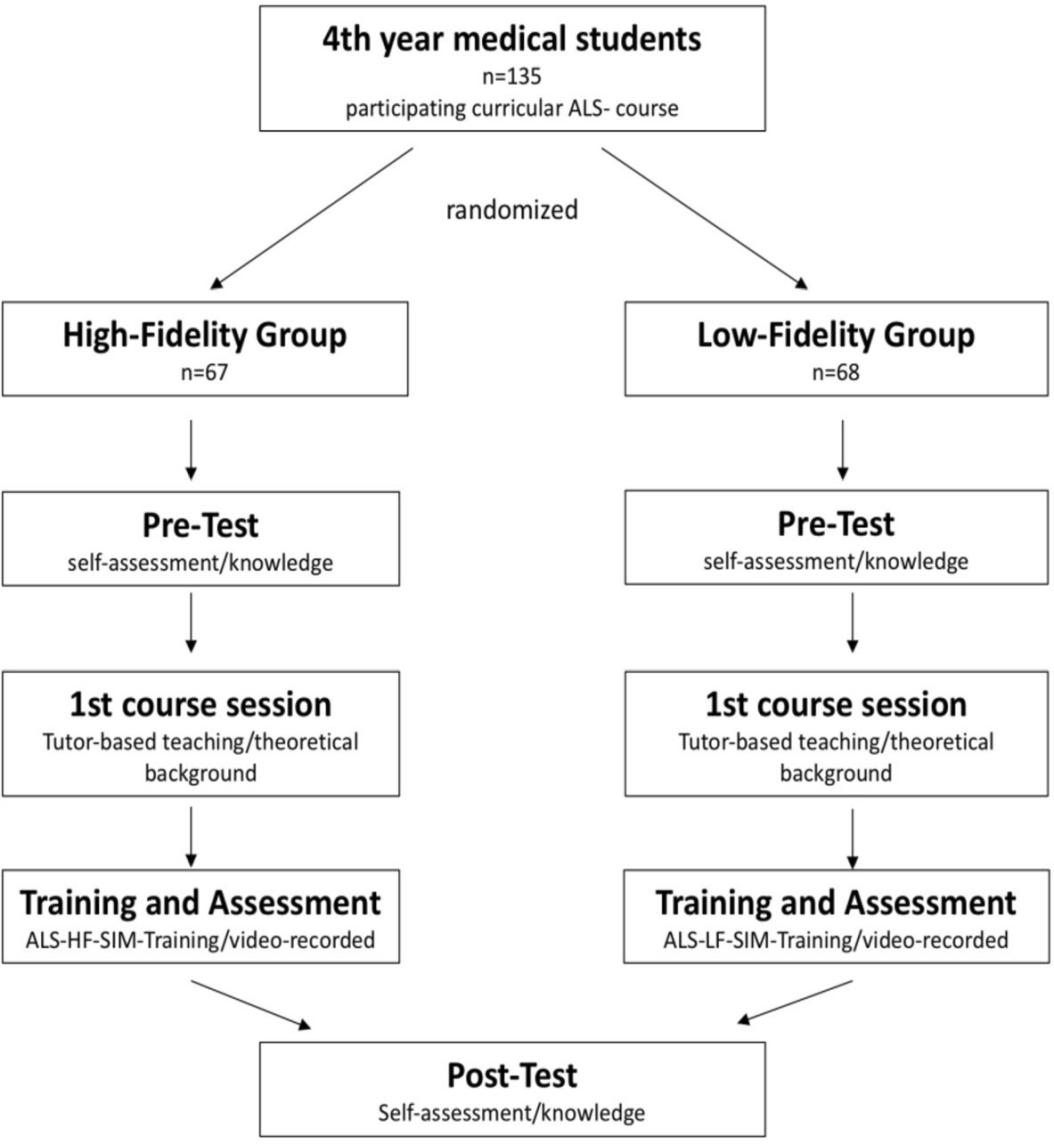
Flowchart

A 20-item multiple choice test with knowledge-based questions, derived from the guidelines of ALS of the European Council of Resuscitation, was used to record students’ knowledge prior to the course. A self-assessment questionnaire comprising 8 items, of which 6 used 10-point Likert scales (ranging from 0 [very poor] to 10 [excellent]) to evaluate knowledge, skills and self-confidence of different qualities, as well as a self-rating against the other group, was completed by each student before the course. Demographic data were recorded. After participation in the course session, self-assessment and 20- item multiple choice questionnaires were conducted again. (Fig. 1).

### Setting of the course session and video analysis

At the beginning of the course session both groups received tutor-based education, using either the LF or the HF mannequin, as per group allocation. Courses for both groups were identical with regards to teaching content. The teaching environment for the HF group scenario took place in a simulated intensive care unit room. A HF patient simulator “SimMan 3G” (Laerdal Medical GmbH Puchheim) was used – this produced effects such as spontaneous breathing with chest excursions and breathing noise, palpable pulses, cyanosis, pupil reaction, a measurable blood pressure and a mannequin-generated voice. The LF group trained in the setting of a regular hospital ward room on a standard “Rescue Anne Simulator” (Laerdal Medical GmbH Puchheim), which features simulated spontaneous breathing and vital signs.

Assessment scenarios took place during the second part of the course. Groups of students had to deal independently with a case of ventricular fibrillation. Four students at a time were asked to apply their previously acquired knowledge of ALS. Tools available in the setting were a defibrillator, ventilation equipment and various intravenous medications. A full-length video of the simulation scenario was recorded. Video analysis was performed and rated by two independent investigators, according to a predefined score sheet. All students received debriefing afterwards.

### Main outcome measures

The primary outcome of the study was the difference between the HF and LF-group in self-assessment. To that end, results of 10-point Likert scaled questions were compared between the study groups before and after the simulation scenario.

Secondary endpoints were the differences in practical performance in the assessment scenario and the growth in theoretical knowledge after the HF or LF-training, respectively.

It was hypothesised that students in the HF-group would rate themselves as superior in comparison to the LF group.

### Statistical analysis

IBM-SPSS (IBM, v23.0) was used for statistical analysis. ANOVA was performed for analysis of self-assessment and multiple-choice examinations. A *t*-test was used on the results of multiple-choice test. The results of the self-assessment were analysed performing Chi-squared test and McNemar’s test on paired samples. Chi-Squared and *t*-tests were applied to the data from the video analysis. Statistical significance was considered at *p* < 0.05.

## Results

### Demographic data

A total of 135 medical students were included in the study and randomly allocated to a HF (*n* = 67) or a LF (*n* = 68) group. Seventy-five (55.6%) were female (HF: 50.7%, LF: 60.3%, Chi-Square test: 1.24, *p* = 0.26) and the mean age was 24 ± 2.9 years in the LF and 23.7 ± 2.8 years in the HF group. No significant differences in demographic data were seen. (Table 1).

**Table 1:** Demographic data

Variable	Group	Mean	SD	Percentage	Chi-Square	*P*-Value
Sex (female)					1.24	0.26
	LF			60.3%		
	HF			50.7%		
Age						0.36
	LF	24	2.9			
	HF	23.7	2.8			
Semester						0.38
	LF	7.0	0.2			
	HF	7.1	0.4			
Previous ALS experience					2.0	0.59
	LF			97%		
	HF			100%		
Previously worked with or for emergency services					0.29	0.59
	LF			11.8%		
	HF			9%		

### Knowledge test

**Fig. 2:**
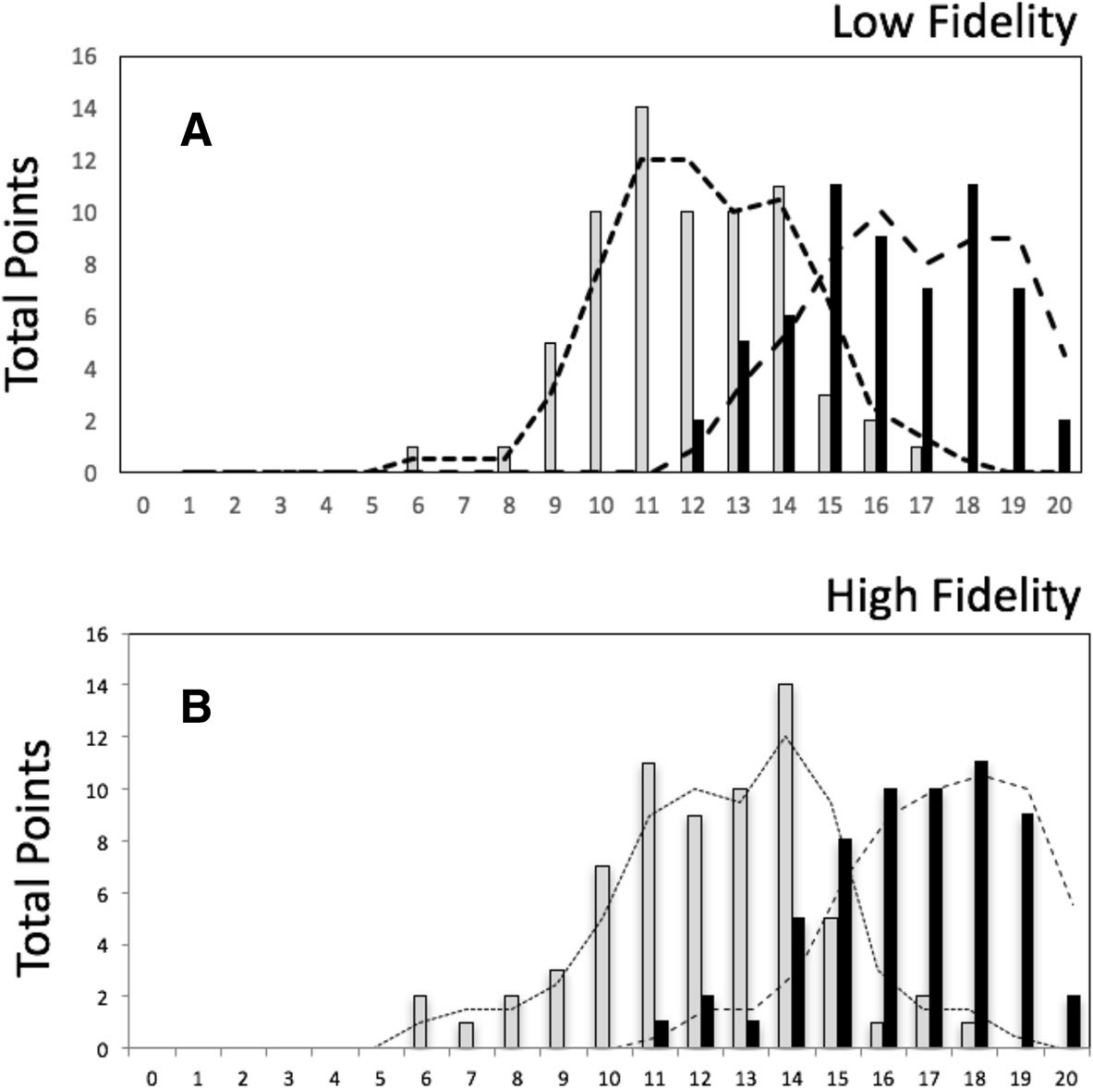
Score distribution in theoretical knowledge pre- (grey) and post-test (black). Both low fidelity group (**a**) and high fidelity group (**b**) improved their theoretical knowledge significantly (*p* < 0.001) but there was no significant intergroup difference

Mean scores in the multiple-choice knowledge pre-test were 12 ± 2.5 (HF) and 11.5 ± 2.1 (LF) and increased to a score of 16.5 ± 2.0 (HF) and 16 ± 2.6 (LF) in the post-test. Both groups improved their theoretical knowledge significantly (*p* < 0.001), however, no significant intergroup differences were detected (*p* > 0.05) (Fig. 2).

### Video analysis

Video analysis and scoring of practical performance resulted in comparable findings for both groups for most evaluation criteria. With regard to “breathing control” (*p* = 0.012), “continuous chest compression while charging the defibrillator” (*p* = 0.017), “electrocardiogram analysis” (*p* = 0.021) and “time interval between electric shocks” (*p* = 0.016), students in the LF group performed significantly better (Table 2).

**Table 2:** Results and group differences from the video analysis

Item	Low-Fidelity	High-Fidelity	*P*-Value
Duration of examination of vital signs	7 s	7 s	
Interval between taking vital signs and chest compression	8 s	6 s	
Adressing the patient	85%	77%	
Pain stimulus	77%	67%	
Examination of breathing			
-not at all	5%	20%	*(*p* = 0.012)
-incorrect examination	69%	62%	
-correct examination	26%	18%	
Call for help	65%	61%	
Time until start of chest compression	22 s	20s	
Time until ventilation	62 s	61 s	
Ventilation without equipment	29%	41%	
Time until defibrillator patches applied	91 s	105 s	
Heart Rhythm assessed	69%	51%	*(*p* = 0.021)
Rhythm assessed correctly	100%	92%	
Continuous chest compression during rhythm assessment	31%	34%	
Time to first defibrillation	151 s	154 s	
Time between application of defibrillator patches to first defibrillation	59 s	59 s	
Mean number of given shocks	2	2	
Time between defibrillations	106 s	91 s	*(*p* = 0.016)
Incorrect application of medication	10%	15%	
placement of a venous cannula	26%	39%	
30:2 ratio compliance	87%	87%	
Disruption of compression >10s	44%	51%	
Guedel oropharyngeal airway used	10%	7%	
Continuous compression during preparation of defibrillator	42%	21%	*(*p* = 0.017)
Pulse palpation	0%	5%	
Intubation	2%	3%	

Significant differences are marked with*

### Self-assessment

**Fig. 3:**
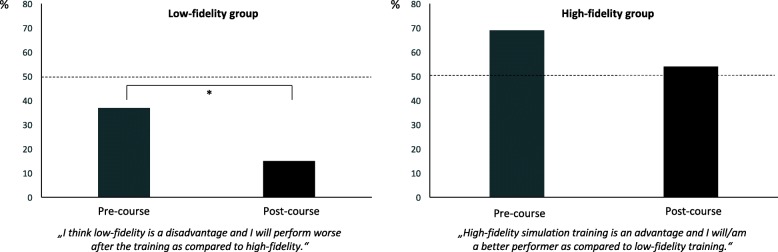
Before (grey) and after (black) course assumptions regarding individual learning success in the low- and high-fidelity groups. Significant differences are marked with*

Before the course (69%) the majority of students in the HF group assumed that they had a significant advantage with regards to their individual learning success, compared to students in the LF group. Participation in the course had no effect on this assumption: 53% of students in the HF group were still convinced of their individual benefit. The difference between pre- and post-course was not statistically significant (*p* = 0.052). In contrast, we found a significant reduction concerning their fear of being disadvantaged between pre-course (37%) and post-course (13%) (*p* = 0.003) in the LF group (Fig. 3).

After the course, 41% of students in the HF group considered themselves to be better performers in handling a resuscitation compared to students in the LF-group, despite their not having witnessed the performance of participants in the LF group.

Before participation in the training, but after group allocation, 84% (HF group) versus 69% (LF group) of students believed there was a positive correlation between the extent of the technical features in the simulator and learning success; and considered HF simulation to be the preferred learning method (*p* = 0.038). After the course, 88% of students in the HF group still considered HF simulation to be the superior learning method and thus recommended it for future training and education, but only 38% of students in the LF group still held that opinion (*p* < 0.001).

## Discussion

Simulation-based training has evolved into an indispensable tool in medical education. Driven by a ‘higher-faster-further’ attitude, the spread of relatively costly and staff-intensive HF simulators has been extensive in recent times [19]. However, whether this development has been reasonable remains unclear. After initial enthusiasm that stemmed from positive early studies, there is now an increasing body of evidence based on the results of high quality randomized controlled trials, comparing various end-points such as knowledge or skill acquisition, that high- or low-fidelity simulation training results in equivalent effects [11–13, 20–23].

Similarly in this study, although training improved both theoretical knowledge and the practical skills of participants in both groups, there was no significant difference between the two methods of training. Interestingly, the LF group performed even better in some of the sub-items. Nonetheless, HF simulators remain popular devices and there are also numerous trials in their favor [24–26].

Using the framework of the classical Miller pyramid of clinical competence assessment with its ascending levels from knowledge at the base to action at the top (knows- > knows how- > shows how- > does) [27], a relationship between specific forms of simulation training and correspondingly achievable levels of competency has been described. However, even if a high degree of realism favours the acquisition of the highest level of the pyramid (“does”-action) it requires for the condition of more qualified trainees proportionally to the degree of realism, to surpass the level of “shows how” [28]. Conversely, this may suggest that trainees at a lower educational level, as medical students in particular, are more likely to benefit from lower degrees of simulation training, supporting the results from this study.

As the evaluation of learning success is mostly performed using pre- and post-tests, with the addition of expert scores, these assessments are particularly hard to blind. A degree of bias cannot be ruled out, since most studies on HF simulators are conducted by investigators who are themselves operating costly simulation centers.

For example, a study by Conlon et al. compared low-, mid- and high-fidelity training groups and found no differences with respect to the improvement of theoretical knowledge, but in the evaluation of practical performance, as scored by expert assessors, the HF group achieved significantly better outcomes [26].

Furthermore, a potential bias favoring HF simulation-based education may emerge from the age of the participants- the higher technical affinity and preference for a technology-based learning approach of millennials as digital natives has been described previously [29]. Also, this type of education and training provides a highly entertaining and positive emotional experience [30–32].

In this study, we found a strong positive expectation favoring the value of HF simulation in most students from both groups before the beginning of the course session, whereas afterwards only a majority of the HF group maintained this belief, while many participants in the LF group had changed their opinion and did not consider LF training to be inferior.

Basak et al. described significantly higher satisfaction scores and a more positively rated experience in a population of medical students practicing on a HF mannequin rather than among a LF simulation control group [32]. McConnell and Eva postulated that the current emotional state of participants had clear implications for the perception and learning of new content [33]. An association between positive emotion, particularly fun, and cognitive capacity, was demonstrated by Duque et al. (2008) [34].

We believe it is likely that emotions influence self-assessment and self-confidence during simulation training. In our trial, participants using HF simulator-training were prone to overrate their abilities and performance, despite showing similar outcomes in theoretical testing and similar, or even inferior, performance of practical skills. This can be considered as misaligned self-awareness, induced by overconfidence in the HF simulator. Participants in the HF group gained increased confidence, without an equivalent increase in knowledge or skill, which contrasted with those in the LF simulation group who provided more realistic self-evaluations.

The ability of medical students to self-assess is known to be limited even though it seems to improve in accuracy in later years of medical school [35]. However, inaccuracy of self-assessment is not only a problem of medical students: irrespective of the level of training the relationship between self-assessment and external assessment was found to be weak [36] Further, self-rated assessment is not only an inappropriate predictor of actual performance [37], those who self-assess more inaccurately are also more likely to perform weakly [36, 38, 39].

Our results confirm in addition that HF simulation training may be associated with mismatching of self-confidence in actual clinical performance. A previous trial comparing simulation-based education with problem-based discussion found that overconfidence was an adverse effect of simulation-based training [14]. This investigation suggests a relationship between the degree of realism in a simulated scenario and the risk of overconfidence. The experience of HF simulation training appears to be associated with a positive emotional state that might induce misconceptions about performance.

Studies in economics have described a positive link between overconfidence and risk-taking behavior in financial professionals [15, 16]. Several medical trials have identified overconfidence as one of the most common cognitive biases leading to diagnostic errors [17, 18]. Although comprehensive studies are still lacking [18], a negative impact on patients’ outcomes is likely, making overconfidence a potential hazard.

An improvement in confidence in general as a result of HF simulation training has been described [32, 40], but data regarding the extent of the increase, in comparison to a control group, are lacking.

### Limitations

Our study has several limitations: Firstly: we conducted the study with a cohort of 4th year medical students. Their particular level of training and education might have affected the results in a different way than an assessment of medical professionals would have had. Further, tutors and raters of the video analysis were aware of the group allocation. This may have had an unintended influence on the teaching style and the review of the assessment performance. Also, as confidence was self-rated, this may have placed more emphasis on the anticipated advantage of HF-training in participants.

## Conclusions

High-fidelity simulation-based education is a highly acclaimed but expensive and resource- intensive method. This study supports the contention that no advantage in learning success is achieved by a higher degree of realism of the simulator. Overall expectations concerning experience and learning outcomes were higher in the HF simulation environment than in the LF setting. A cognitive bias towards highly realistic, technically well-equipped learning tools is probable. Participation in the HF simulation led to misconceived self-assessments in terms of actual abilities and consequently overinflated self-confidence. Being potentially associated with the specific educational level of our cohort, it remains unclear whether our results are also transferable to medical professionals. Future research is required, as it remains questionable whether the additional costs and expenses for HF simulators are justified if only comparable knowledge and skill outcomes are achieved. This is all the more so if undesirable effects such as excessive self-confidence contribute to flawed decision-making, with the potential for worse patient outcomes. Or as Oscar Wilde famously noted, “Confidence is good, but overconfidence always sinks the ship.”
